# The Histology and Histochemistry of Man

**Published:** 1875-10

**Authors:** 


### BIBLIOGRAPHICAL.

*The Histology and Histochemistry of Man.*—A Treatise on the Elements of Composition and Structure of the Human Body. By Heinrich Frey, Professor of Medicine in Zurich. Translated from the Fourth German Edition by Arthur E. J. Baker, Surgeon to city of Dublin Hospital; Demonstrator of Anatomy Royal College of Surgeons, Ireland ; etc., etc., etc., and revised by the Author. D. Appleton & Co., Publishers, New York, 1875.

This is a very comprehensive and elaborate treatise, which has become the standard work of Pathological Histology ; the aim of the author being to vindicate the dignity of this science and demonstrate its most elementary principles. That he has succeeded in presenting a perfect compendium of the views of the most reliable and thorough histologists this work is an evidence; and all interested in the subject will find it to be a valuable contribution to medical literature. The contents present first, the Albuminous or Protein Compounds of the Elements of Composition and Structure of the Body, Hæmoglobulin, Histogenic Derivatives of the Albuminous Substances or Albuminoids, the Fatty Acids and Fats, the Carbohydrated, The Non-nitrogenous Acids, The Nitrogenous Acids, Amides, Amido Acids and Organic Bases, Animal Colouring Matters, Cyanogen Compounds, Mineral Constituents. Second: The Tissues of the Body—Tissues Composed of Simple Cells with Fluid Intermediate Substance, and also of Solid Intermediate Substances, and those belonging to the Connective Substance Group, those composed of Transformed Cohering Cells and Composite Tissues. Third: The Organs of the Body—Organs of the Vegetative Type, and of the Animal Group. The work is illustrated with six hundred and eighth wood engravings, and contains six hundred and eighty-three pages,

